# Alterations of Both Dendrite Morphology and Weaker Electrical Responsiveness in the Cortex of Hip Area Occur Before Rearrangement of the Motor Map in Neonatal White Matter Injury Model

**DOI:** 10.3389/fneur.2018.00443

**Published:** 2018-06-19

**Authors:** Yoshitomo Ueda, Yoshio Bando, Sachiyo Misumi, Shino Ogawa, Akimasa Ishida, Cha-Gyun Jung, Takeshi Shimizu, Hideki Hida

**Affiliations:** ^1^Department of Neurophysiology and Brain Science, Nagoya City University Graduate School of Medical Sciences, Nagoya, Japan; ^2^Department of Functional Anatomy and Neuroscience, Asahikawa Medical University, Asahikawa, Japan; ^3^Department of Obstetrics and Gynecology, Nagoya City University Graduate School of Medical Sciences, Nagoya, Japan

**Keywords:** hypoxia-ischemia in premature infants, white matter injury, intracortical microstimulation (ICMS), golgi staining, cortical layer I-III, hip area, dendritic branches

## Abstract

Hypoxia-ischemia (H-I) in rats at postnatal day 3 causes disorganization of oligodendrocyte development in layers II/III of the sensorimotor cortex without apparent neuronal loss, and shows mild hindlimb dysfunction with imbalanced motor coordination. However, the mechanisms by which mild motor dysfunction is induced without loss of cortical neurons are currently unclear. To reveal the mechanisms underlying mild motor dysfunction in neonatal H-I model, electrical responsiveness and dendrite morphology in the sensorimotor cortex were investigated at 10 weeks of age. Responses to intracortical microstimulation (ICMS) revealed that the cortical motor map was significantly changed in this model. The cortical area related to hip joint movement was reduced, and the area related to trunk movement was increased. Sholl analysis in Golgi staining revealed that layer I–III neurons on the H-I side had more dendrite branches compared with the contralateral side. To investigate whether changes in the motor map and morphology appeared at earlier stages, ICMS and Sholl analysis were also performed at 5 weeks of age. The minimal ICMS current to evoke twitches of the hip area was higher on the H-I side, while the motor map was unchanged. Golgi staining revealed more dendrite branches in layer I–III neurons on the H-I side. These results revealed that alterations of both dendrite morphology and ICMS threshold of the hip area occurred before the rearrangement of the motor map in the neonatal H-I model. They also suggest that altered dendritic morphology and altered ICMS responsiveness may be related to mild motor dysfunction in this model.

## Introduction

Although advances of perinatal medicine have improved the survival rate of preterm infants (1, 2), these infants often have neurological insults due to hypoxia-ischemia (H-I) accompanied with brain immaturity (3). A profound shift in the features of H-I over time has also been reported: a milder form of H-I, characterized by nondestructive lesions, is reportedly increasing in prevalence (4–7). However, there is a pressing need to expand current understanding of the mechanisms underlying the effects of brain insult and to develop treatments for the resulting behavioral and cognitive dysfunction in development.

Preterm infants have a higher risk of neonatal white matter injury (WMI) because late oligodendrocyte progenitor cells (OPC), which are abundant at gestational weeks 20–28 in humans (8), are particularly susceptible to H-I (9–11). Ischemia-induced neuroinflammation and prenatal inflammatory response are known to be related to neonatal WMI (12–15). Neonatal WMI causes neurodevelopmental deficits during development, including motor deficits (such as cerebral palsy), learning disorders, and behavioral difficulties (including attention deficit/hyperactivity disorder) (16–18). Various animal models of preterm infants have been reported, including models using sheep (19, 20), rabbit (21–23), piglets (24–26), and rodents (15, 27–32). Among the available rodent models, the Rice-Vannucci model (33) and its variations have been commonly used (27, 28, 30). Other models, created by unilateral uterine artery ligation of dams at embryonic day (E) 17 (34, 35) or transient bilateral occlusion of the uterine arteries at E18 (15, 29, 31), have also been reported.

We previously established a rat model of neonatal WMI produced by right carotid artery occlusion followed by under 6% oxygen for 1 h (36), based on the notion that late development of OPC (preOLs) in the immature brain is specifically associated with vulnerability to H-I (11). In this neonatal WMI model, actively proliferating OL progenitors are primarily damaged, with a decreased number of mature OL cells and hypomyelination in the sensorimotor cortex in adulthood, indicating that impaired motor coordination is induced by impaired myelination in layer I-IV rather than neuronal loss (37). This neonatal WMI model exhibited moderate motor deficits, especially in the hindlimbs, accompanied with disorganization of oligodendrocyte development in layers II–III of the sensorimotor cortex (38).

As neonatal WMI is a complex amalgam of destructive developmental disturbances in premature infants (5), changes in neuronal circuit formation and connectivity in the cerebral cortex may be affected by the close relationship between neuronal development and myelination. Although our model has revealed motor coordination dysfunction without the loss of cortical neurons (37, 38), it remains unclear how motor dysfunction is induced in this model.

To reveal the mechanisms underlying imbalanced motor coordination in the neonatal WMI model, we investigated electrical responsiveness, and dendrite morphology in the sensorimotor cortex in adulthood. Thus, intracortical microstimulation (ICMS) in the hindlimb motor cortex was performed to reveal electrophysiological responses, and morphological changes were examined in the motor cortex using Golgi staining.

We found that the cortical motor map was significantly changed in neonatal WMI model at 10 weeks of age: the cortical area related to hip joint movement was reduced while the area related to trunk movement was increased. In addition, we found that layer I–III neurons on the H-I side had more dendrite branches compared with the contralateral side in Golgi staining. ICMS and Golgi staining performed at 5 weeks of age revealed that the minimal ICMS current to evoke twitches was higher on the H-I side while the motor map was unchanged, and more dendrite branches in layer I–III neurons were shown in the H-I side, indicating that alterations of both dendrite morphology and ICMS threshold of the hip area occurred before the rearrangement of the motor map in the neonatal H-I model.

## Materials and methods

### Animals

Animal care was carried out according to the guidelines of the Institute for Experimental Animal Sciences, Nagoya City University Graduate School of Medical Sciences, Nagoya, Japan. All experimental procedures were approved by the committee of animal experimentation of Nagoya City University Medical School, and appropriate measures were taken to minimize the pain and discomfort of the animals used in the study. Until weaning, 10 male Wistar rat pups (Japan SLC, Japan) were reared with a foster mother. After weaning, 3–5 rats were housed together in each cage. A total of 48 rats from multiple different litters were euthanized for this study, including 17 rats as controls, and 31 rats as neonatal H-I models.

### Neonatal WMI model

A procedure involving H-I treatment was conducted to produce neonatal WMI model, as we confirmed many characteristics of the injury in the white matter in our previous papers (36–38). Rat pups at postnatal day 3 (P 3) were subjected to right common carotid artery cauterization under isoflurane (Pfizer, NY, USA) anesthesia $(5% [v/v] induction, 1.0% [v/v]) and kept at 37°C on a heat pad. After a 2-h recovery period with their dam, pups were exposed to 6% (v/v) O_2_ hypoxia for 60 min in a container submerged in a 38°C water bath.

As a control condition, we performed 6% hypoxia after a sham operation: skin incision, separation around the right common carotid artery and skin suture were performed before the hypoxia. We used this condition as controls for ICMS because we previously confirmed that there was no significant difference between “sham operation with normoxia” and “sham operation with 6% hypoxia” in histology (the numbers of neurons and microglia, the morphology of microglia, and the intensity of myelin basic protein staining) or behavior (motor function by motor deficit score) (38).

### Intracerebral microstimulation (ICMS)

We first performed ICMS at 10 weeks of age to investigate differences in the pattern of motor maps (*n* = 9 for neonatal WMI and *n* = 8 for controls). ICMS was then performed at 5 weeks of age to reveal the developmental pattern of the change (*n* = 11 for neonatal WMI and *n* = 9 for controls), using a procedure similar to methods described elsewhere (39–41) with some modifications (42).

Rats underwent anesthesia with a mixture of ketamine (60 mg/kg, i.p.) and xylazine (10 mg/kg, i.p.), and dexamethasone (0.1 mg/kg) was then administered into the right gluteal fascia to prevent brain edema. Thirty minutes after the first injection, a mixture of ketamine (30 mg/kg) and xylazine (5 mg/kg) was injected into the right gluteal fascia to maintain constant anesthesia during cranial scraping. Supplemental injection of ketamine (40 mg/kg), or a mixture of ketamine (15 mg/kg) and xylazine (2.5 mg/kg), was occasionally used when animals exhibited vibrissae twitching, muscle twitching and/or tachypnea during recording, followed by a 10-min break from recording to keep the measurement consistent.

Rats were fixed on a stereotaxic apparatus (Narishige, Japan), and craniotomy was performed to expose the right sensorimotor cortex. The coordinates for the openings were 1.0–3.0 mm posterior and 1.0–3.0 mm lateral from the bregma, known as the sensorimotor area, including the hindlimb area. The exposed area was regularly spaced out over a 0.5-mm grid. Under a microscope, a glass-insulated tapered tungsten electrode (250 μm shank diameter, impedance, 0.7 MΩ; Alpha Omega #380-080607-11, GA, USA) was lowered perpendicularly into the cortex to 1,600 μm below the cortical surface, a depth corresponding to layer V of the sensorimotor cortex. The electrode was then adjusted by ±200 μm to find the appropriate location for measuring the spike pattern with an oscilloscope and loudspeaker. Thirty biphasic pulses (333 Hz, 200 μs pulse duration) produced by an electrical stimulator (Nihon Kohden, Japan) connected to an isolator (Nihon Kohden, Japan) were passed through the electrode every 2 s, and the response of the cortex was amplified using an amplifier (AM Systems, WA, the USA) connected to an oscilloscope. Starting at a current of 0 μA, intensity was increased in 10 μA steps until twitching of the hip joint, knee joint, foot joint, digit and trunk were confirmed by palpation and/or visual inspection. The current was then gradually decreased until the twitch was no longer detectable. This level was defined as the current threshold. If no twitches were evoked at 200 μA, the site was defined as “non-responsive.”

**Figure 1:**
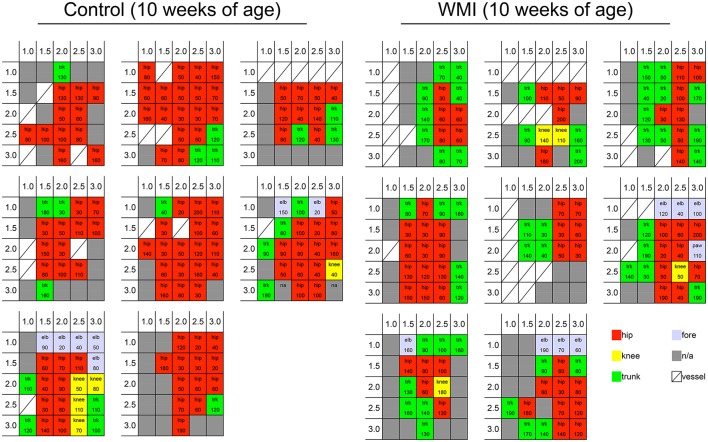
Cortical motor map by ICMS at 10 weeks of age. We stimulated the sensorimotor cortex per 0.5 mm at 1.0–3.0 mm right and 1.0–3.0 mm posterior from the bregma. Each grid shows a cortical map of each rat cortex in control group (*n* = 8) and neonatal WMI group (*n* = 8). Blocks in the grids show the portion of the rat body in which twitching was observed (hip joint, knee joint, trunk, and forelimb), and the current threshold is presented in each grid. Cortical maps are shown as a 5-colored code in the grid: twitching of the hip joint (red), knee joint (yellow), foot joint (light blue), and trunk (green), and non-responsive square (gray).

**Figure 2:**
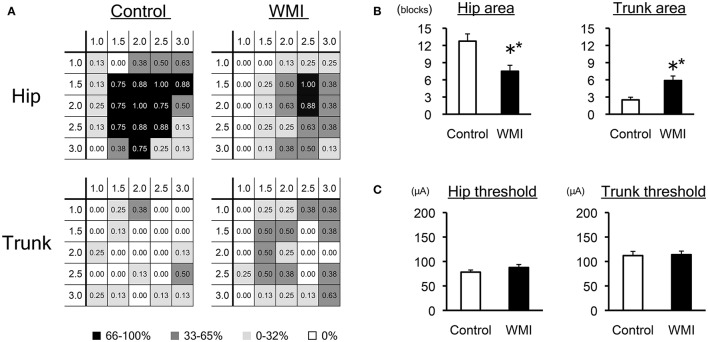
Electrophysiological analysis of sensorimotor cortex by ICMS at 10 weeks of age**. (A)** A summary of the motor maps in Figure 1 is shown as a heat map that represents the occurrence rate of each stimulated spot: the number of animals in which the movement was elicited was taken over the number of times that spot was stimulated in each group (*n* = 8 for each group). Each number in the grid shows the rate of responsive individuals in each portion, displayed as monochrome heat maps. Note that the rate for the hip area decreased in the WMI model, whereas the rate for the trunk area increased. **(B)** The number of grids associated with the hip area was significantly reduced, while that of the trunk area significantly increased in neonatal WMI model animals. **(C)** The minimal current intensity (threshold) to evoke muscle twitch was unaltered between the groups with regard to both the hip and trunk area. **p < 0.01 by Mann–Whitney *U*-tests. Data are presented as mean ± SEM.

ICMS data are represented as cortical maps with current thresholds in Figure 1, (Supplemental Figure 1, and as heat maps in Figures 2A, **4A**. Cortical maps were created using areas that responded to the stimulation and are shown as 5-colored code in the grid: in each animal, we noted for each stimulated square whether the stimulation resulted in twitching of the hip joint (red), knee joint (yellow), foot joint (light blue), and trunk (green), or the non-responsive square (gray). The generated heat maps represent the occurrence rate of each stimulated spot in the sensorimotor area, and were calculated separately for each experimental group: for each point, the number of animals in which the movement was elicited was taken over the number of times that spot was stimulated in each group. For example, if three animals in a group responded to cortical stimulation at a cortical spot when five of the animals in the group were stimulated at that point, the occurrence rate would be 0.6. To visualize the organization of the cortical spots with higher occurrence rates of the movements, a 4-grade white-black code was applied to the map.

### Golgi staining

We used an FD Rapid GolgiStain Kit (COSMO BIO, Japan) for Golgi staining according to the standard protocol (43). The rats were deeply anesthetized with sodium pentobarbital, and cervical dislocation was performed. Coronal brain sections of 2 mm thickness (1.0–3.0 mm posterior to the bregma, which was the same area used for ICMS) were obtained using a brain matrix and soaked in Golgi-Cox solution for 14 days followed by 30% sucrose for 3 days. The sections were sliced into 200 μm, stained and photographed with BZ-X700 (Keyence, Japan) focusing on the motor cortex (same area for ICMS), particularly layers I–III.

Based on our previous paper (44), we used Sholl analysis to evaluate dendrite expansion from a cell body: the numbers of cross sections at every 20-μm from center of the cell body were counted in 27 Golgi-positive neurons per animal (neonatal WMI model: *n* = 4 at 5 weeks of age and *n* = 5 at 10 weeks of age; sham-operated control: *n* = 1).

### Immunohistochemistry for microglia

We performed immunohistochemistry at P 17 (*n* = 4) and P 28 (*n* = 4). Under deep anesthesia with pentobarbital (>50 mg/kg), rats were perfused with 4% paraformaldehyde in 0.1 M phosphate buffered saline (PBS). The brains were obtained, post-fixed with the same fixative overnight, and cryoprotected with 30% sucrose. Coronal-sections (40 μm) were prepared from 1–3 mm posterior to the bregma that contains the sensorimotor cortex of the hindlimb area. After soaking with 0.25% Triton X-100 in PBS (PBS-T), blocking was performed with 10% normal goat serum (NGS) (Vector Labs, USA) for 60 min. The slices were reacted with anti-Iba1 polyclonal antibody (1:1,000; Wako, Japan) immersed in PBS-T containing 1% NGS at 4°C overnight, followed by immersion goat anti-rabbit IgG conjugated with Alexa Fluor 594 (1:1,000; Abcam, UK). After the slices were embedded on a glass slide with mounting medium (Vector Labs, USA), slices were photographed with a fluorescence microscope (Axio Observer.Z1; Zeiss, Germany) and an AX70 microscope (Olympus, Japan).

### Statistics

Mann–Whitney *U*-tests were used to compare the current threshold and the number of blocks between control and neonatal WMI rats in the ICMS experiment. Mann–Whitney *U*-tests were also used to compare the number of cross sections between ipsilateral side and contralateral side in Scholl analysis. All data are shown as mean ± standard error of the mean (SEM).

## Results

### Motor map change of the neonatal WMI model in adulthood at 10 weeks of age

To examine neuronal responsiveness in the sensorimotor cortex, we performed ICMS focusing on the right H-I side compared with the right control side at 10 weeks of age (Figures 1, 2). We stimulated the hindlimb area of the sensorimotor cortex because gross hindlimb function exhibited more disruption than forelimb function in our previous study (37, 38).

Each cortical map in the control group (*n* = 8) and neonatal WMI group (*n* = 8) is shown in Figure 1. To clarify the map expansion of each group, we re-organized the map data into heat maps of the hip and the trunk (Figure 2A). The heat maps clearly revealed a decrease in the size of the hip area and enlargement of the trunk area in the WMI group. The number of blocks in the hip area and the trunk area confirmed a significant decrease in the hip area (control: 12.8 ± 1.3, *n* = 8; WMI: 7.5 ± 1.0, *n* = 8; *p* < 0.01) and a significant increase in the trunk area (control: 2.5 ± 0.5, *n* = 8; WMI: 5.9 ± 0.8, *n* = 8; *p* < 0.01) (Figure 2B). In contrast, the current threshold for evoking the movements remained unchanged in both the hip joint (control: 78.2 ± 4.3 μA; WMI: 87.6 ± 6.2 μA) and trunk muscle (control: 112 ± 8.57 μA; WMI: 114 ± 7.26 μA) (Figure 2C).

### Change of dendrite expansion in the neonatal WMI model in adulthood

**Figure 3:**
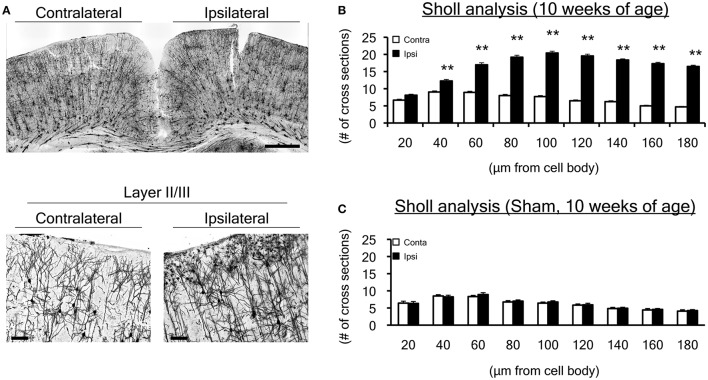
Dendrite morphological changes in the motor cortex at 10 weeks of age. **(A)** We performed Golgi staining on brain slices, focusing on the hindlimb motor cortex. We particularly focused on layers I-III, in which histological changes were observed in our previous study. **(B)** To evaluate dendrites projecting from a cell body, a total of 135 Golgi-positive neurons in both ipsilateral and contralateral sensorimotor cortex were assessed by Sholl analysis in the neonatal WMI model (*n* = 5) at 10 weeks of age. Sholl analysis revealed that the number of cross sections increased over 40 μm from cell bodies on the right (ipsilateral H-I) side of the cortex. **(C)** Dendrite projections of 27 Golgi-positive neurons in the sham-operated right sensorimotor cortex were similar to those in the left cortex, which was equivalent to the left (contralateral control) side of the WMI group in B. **p < 0.01 by Mann-Whitney *U*-tests. Scale bar, 100 μm in lower figures. Data are presented as mean ± SEM.

To investigate whether neuronal morphology changed at 10 weeks of age, when cortical map reorganization was altered, we performed Golgi staining focusing on the same sensorimotor cortex in neonatal WMI rats (*n* = 5) (Figure 3). To evaluate developmental changes in morphology, we used Sholl analysis and counted the number of cross sections every 20-μm from the center of the cell body (Figure 3B). Neurons in the right H-I hemisphere (ipsilateral side) appeared to exhibit denser expansion of dendrites in layers I–III (Figure 3A). A similar pattern of dendrite expansion was observed in layer V of the neonatal WMI model (data not shown), although accurate analysis was difficult due to the higher neuron density. Sholl analysis revealed that neurons on the H-I side had significantly more dendritic branches (Figure 3B; Supplemental Table 1). In contrast, sham-operated animals exhibited no differences between hemispheres (Figure 3C), showing an equivalent number to the contralateral side of the neonatal WMI model (data for the contralateral side in Figure 3B).

### Electrophysiological and morphological changes in earlier developmental stages at 5 weeks of age

To determine whether the electrophysiological and morphological changes described above were exhibited at earlier developmental stages, we also performed ICMS and Golgi staining at 5 weeks of age.

**Figure 4:**
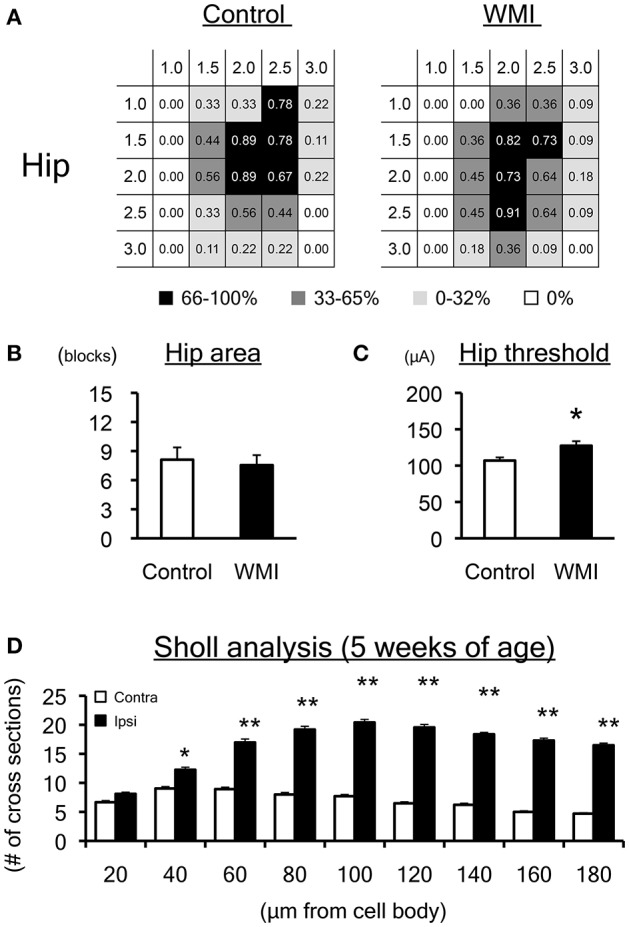
Motor map and dendrite expansion at 5 weeks of age. **(A)** A summary of the motor maps in Supplemental Figure 1 is shown as a heat map. Although map size at 5 weeks of age appears to be smaller than that at 10 weeks of age, the size of the hip area of the WMI group (*n* = 11) was unchanged compared with control animals (*n* = 9). **(B)** The number of blocks in the hip area was unaltered between groups. **(C)** The threshold for eliciting hip joint twitch was significantly higher in the WMI group. **(D)** Twenty-seven Golgi-positive neurons per animal were assessed by Sholl analysis in both ipsilateral and contralateral sensorimotor cortex of neonatal WMI model (*n* = 4) at 5 weeks of age. Sholl analysis revealed that the number of cross sections increased over 60 μm from cell bodies on the right side of the cortex, even at 5 weeks of age. *p < 0.05 and **p < 0.01 by Mann–Whitney *U*-tests. Data are presented as mean ± SEM.

At 5 weeks of age, the map size appeared to be smaller than that at 10 weeks old (Supplemental Figure 1). The heat map indicated that the size of the hip area of the neonatal WMI group (*n* = 11) was unchanged compared with the control animals (*n* = 9) (Figure 4A). The number of blocks in the hip area in each animal was also unaltered between groups (control: 8.1 ± 1.4; WMI: 7.5 ± 0.6) (Figure 4B). In contrast, the threshold of the hip area was significantly higher in the neonatal WMI group (control, 107 ± 6.44 μA; WMI, 127 ± 5.50 μA; *p* < 0.05) (Figure 4C, Supplemental Figure 1). Sholl analysis of layers I–III in the motor area (*n* = 4) indicated that neurons in the right hemisphere had significantly more dendritic branches (Figure 4D, Supplemental Table 1).

It has been previously reported that microglia have a phagocytotic effect on the elimination of synapses during development (pruning) (45–48). To test the possibility that microglial activation was related to changes in cortical map reorganization (ICMS), Iba1 immunohistochemistry was performed at P 17 and P 28 (before 5 weeks old) because morphological changes of the dendrites (Golgi staining) were already observed at 5 weeks of age.

Iba-1 positive cells were detected in the parietal cortex of the neonatal WMI brain (Supplemental Figure 2A). There were many Iba-1 positive microglia with amoeboid bodies and thick processes on the H-I side at P 17 (*n* = 4) (Supplemental Figure 2B). However, Iba-1 positive cells were not detected in the neonatal WMI model at P 28 (*n* = 4) (Supplemental Figure 2C).

## Discussion

To elucidate how motor dysfunction in neonatal WMI is induced without the loss of cortical neurons, we investigated electrical responsiveness and dendrite morphology in the sensorimotor cortex in adulthood (10 weeks of age), as well as earlier developmental stages (5 weeks of age) in WMI model. The results revealed that the motor map in the sensorimotor cortex was altered at 10 weeks of age but not 5 weeks of age. However, the threshold of the hip area was higher on the H-I side at 5 weeks of age, while it was similar at 10 weeks of age. In addition, an increased number of dendritic branches in layers I–III were shown in the H-I cortex as early as 5 weeks old, and this increase was maintained until 10 weeks old. Interestingly, activated microglia were detected on the H-I side at P 17, but not at P 28.

### Alteration of motor maps in the neonatal WMI model

It is known that the cortical motor map changes after brain insults such as intracerebral hemorrhage and spinal cord injury, but can be recuperated by rehabilitation (42) and motor training (40). Environmental enrichment is one method for improving behavioral performance, and has been found to promote cortical development (49). Thus, the motor map is closely related to motor function. These previous findings suggest that the alteration of the motor map at 10 weeks of age found in the present study (hip area reduction and trunk area expansion) may be related to the imbalanced motor coordination in the neonatal WMI model (38). However, alterations of the motor map cannot completely explain the motor imbalance in this model, because it was unchanged at 5 weeks of age in the present study.

A previous study reported that the motor map emerges after 4 weeks old and eventually expands, while gradual decreases of the current threshold are promoted by enriched environments in development (49). These findings indicate that the period after weaning is a critical window in the development of normal function acquisition. Therefore, the higher threshold we detected in the hip area of the WMI group at 5 weeks of age might indicate a developmental delay of functional acquisition, as the threshold became almost equivalent at 10 weeks of age.

### Alteration of dendrite morphology in the surface area of the cortex

Although both excitatory and inhibitory neurons were maintained in the sensorimotor cortex of this model (37), the surface area was affected, exhibiting oligodendrocyte loss and hypomyelination (37, 38). However, it remains unclear how motor dysfunction is induced in this model. Changes in neuronal circuit formation and connectivity in the cerebral cortex could be induced, as neonatal WMI is a complex amalgam of destructive developmental disturbances in premature infants (5). Morphological changes of dendrites in layers II–III were also confirmed by detailed analysis with Golgi staining as early as P 28 in the present study. As the surface area in the sensorimotor cortex receives many afferent fibers, the altered dendritic morphology of Golgi-positive neurons in layers II–III, as revealed by Scholl analysis in this study, might change local neuronal circuits and connectivity, causing the motor imbalance observed in this model.

The current study was unable to provide a clear answer regarding the question of how this morphological change in layers II–III is related to the response to ICMS in this model. GABAergic neurons in the sensorimotor cortex should be taken into consideration, as inhibitory neurons are important for normal motor map function (49), and the motor map changes immediately with injection of a GABA receptor antagonist to the motor cortex (50). Although further studies will be required to understand the contribution of GABAergic neurons, it is important to consider the migration mechanisms and temporal excitatory effects of immature GABAergic neurons by P 14–16.

The retardation of OL differentiation (37, 38) is likely to affect cortical development, as OL-expressing proteins such as myelin associated glycoprotein, Nogo-A, and oligodendrocyte-myelin glycoprotein changed neurite outgrowth during development after neonatal hypoxia (51). Thus, changes of dendrite morphology in layers II–III of sensorimotor cortex in adulthood could be altered by oligodendrocyte loss and hypomyelination (37, 38).

### Possible effects of iba-1 positive microglia

Strong expression of ED1-positive microglial/macrophage cells was detected in the cortex of the model at P 5 in our previous report (36). In this study, the appearance of Iba-1 positive microglia was confirmed in the sensorimotor cortex until P 17, but the cells were unremarkable on both sides of the cortex at P 28.

Previous studies have reported that microglia affect synaptic pruning during development (45–48), and that synapse maintenance and the stripping function of microglia are related to dendrite expansion (52–54). Thus, it is likely that transient appearance of microglial cells until P 28 affected dendritic changes in the cortex that were detected at 5 weeks of age in this study. Further studies will be necessary to examine the morphology during earlier periods of development, and to elucidate the relationship between microglia and dendrite expansion in more detail.

### Limitations of the study

It should be noted that only male pups were used in this study, as there are several reports about the sex-dependent differences of the response to H-I (55–58). Several points in the present study would be strengthened by further experiments as follows. As our discussion related to possible effects of Iba-1 positive microglia is highly speculative, co-staining of Iba-1 with different antigens or synaptic markers, detection of genes related to pro-inflammatory cytokines, and unbiased quantification of Iba-1 positive microglial number (59, 60) should be performed in future. In addition, small sample size of Golgi-staining of sham-operated animal and lack of true control (sham-operation with normoxia) will be solved in near future. As for the limitations in ICMS experiment, the exact motor map boundaries of the hip and trunk are not outlined in this study due to our large regularly space over a 0.5-mm grid and small sample size of our study. It might be possible to get the exact motor map boundaries using more precise ICMS experiment (35).

## Conclusion

The current results demonstrated that the neonatal WMI model exhibited alterations of neuronal morphology and cortical responsiveness. Our data also indicated that alterations of dendrite morphology and electrical responses in the cortex occurred before the rearrangement of the motor map in the neonatal WMI model.

## Author contributions

ICMS was performed my YU with assistance of AI and Golgi staining was performed by YB with hard assessment by YU and SM. The hypoxia-ischchemia model was made by SM with the help by SO, followed Iba1 staining by TS. C-GJ and TS are involved in helpful discussion and joined in writing paper. The draft of this paper was written by YU. HH organizes this study and wrote this paper as correspondence. This study is mainly supported by the grants to SM, YU, and HH with partial support of the grants to C-GJ and TS.

### Conflict of interest statement

The authors declare that the research was conducted in the absence of any commercial or financial relationships that could be construed as a potential conflict of interest.

## Supplementary material

Supplementary Figure 1 Cortical motor map by ICMS at 5 weeks of age. Cortical maps at 5 weeks of age (*n* = 9 for control, *n* = 11 for neonatal WMI) are shown as a 5-colored code in the grid: twitch of the hip joint (red), knee joint (yellow), foot joint (light blue), and trunk (green), and non-responsive square (gray). The current threshold at each point is presented in each grid.

Supplementary Figure 2 Iba1 immunostaining. **(A)** Iba1 immunoreactivity was upregulated on the right H-I side of the cortex at P 17 (*n* = 4). **(B)** Appearance of Iba-1 positive microglia was increased in the right hemisphere (R) compared with the left side (L). **(C)** At P 28 (*n* = 4), the upregulation of immunoreactivity was not seen in either hemisphere. Error bars show 1 mm **(A)**, and 40 μm **(B)**.

Supplementary Table 1 Sholl analysis.

## References

[1] Saigal S Doyle LW. An overview of mortality and sequelae of preterm birth from infancy to adulthood. Lancet (2008) 371:261–9. 10.1016/S0140-6736(08)60136-1 18207020

[2] Costeloe KL Hennessy EM Haider S Stacey F Marlow N Draper ES. Short term outcomes after extreme preterm birth in England: comparison of two birth cohorts in 1995 and 2006 (the EPICure studies). BMJ (2012) 345:e7976. 10.1136/bmj.e7976 23212881 PMC3514472

[3] Rees S Inder T. Fetal and neonatal origins of altered brain development. Early Hum Dev. (2005) 81:753–61. 10.1016/j.earlhumdev.2005.07.004 16107304

[4] Inder TE Anderson NJ Spencer C Wells S Volpe JJ. White matter injury in the premature infant: a comparison between serial cranial sonographic and MR findings at term. Am J Neuroradiol. (2003) 24:805–09. 12748075 PMC7975772

[5] Volpe JJ. Brain injury in premature infants: a complex amalgam of destructive and developmental disturbances. Lancet Neurol. (2009) 8:110–24. 10.1016/S1474-4422(08)70294-1 19081519 PMC2707149

[6] Back SA Rosenberg PA. Pathophysiology of glia in perinatal white matter injury. Glia (2014) 62:1790–815. 10.1002/glia.22658 24687630 PMC4163108

[7] Tusor N Benders MJ Counsell SJ Nongena P Ederies MA Falconer S. Punctate white matter lesions associated with altered brain development and adverse motor outcome in preterm infants. Sci Rep. (2017) 7:13250. 10.1038/s41598-017-13753-x 29038505 PMC5643493

[8] Craig A Ling Luo N Beardsley DJ Wingate-Pearse N Walker DW Hohimer AR. Quantitative analysis of perinatal rodent oligodendrocyte lineage progression and its correlation with human. Exp Neurol (2003) 181:231–40. 10.1016/S0014-4886(03)00032-3 12781996

[9] Back SA Gan X Li Y Rosenberg PA Volpe JJ. Maturation-dependent vulnerability of oligodendrocytes to oxidative stress-induced death caused by glutathione depletion. J Neurosci. (1998) 18:6241–53. 10.1523/JNEUROSCI.18-16-06241.1998 9698317 PMC6793198

[10] Back SA Luo NL Borenstein NS Levine JM Volpe JJ Kinney HC. Late oligodendrocyte progenitors coincide with the developmental window of vulnerability for human perinatal white matter injury. J Neurosci. (2001) 21:1302–12. 10.1523/JNEUROSCI.21-04-01302.2001 11160401 PMC6762224

[11] Back SA Han BH Luo NL Chricton CA Xanthoudakis S Tam J. Selective vulnerability of late oligodendrocyte progenitors to hypoxia-ischemia. J Neurosci. (2002) 22:455–63. 10.1523/JNEUROSCI.22-02-00455.2002 11784790 PMC6758669

[12] Saadani-Makki F Kannan S Lu X Janisse J Dawe E Edwin S. Intrauterine administration of endotoxin leads to motor deficits in a rabbit model: a link between prenatal infection and cerebral palsy. Am J Obstet Gynecol. (2008) 199:651.e1-7. 10.1016/j.ajog.2008.06.090 18845289 PMC2913549

[13] Wang X Stridh L Li W Dean J Elmgren A Gan L Lipopolysaccharide sensitizes neonatal hypoxic-ischemic brain injury in a MyD88-dependent manner. J Immunol. (2009) 183:7471–77. 10.4049/jimmunol.0900762 19917690

[14] Falahati S Breu M Waickman AT Phillips AW Arauz EJ Snyder S. Ischemia-induced neuroinflammation is associated with disrupted development of oligodendrocyte progenitors in a model of periventricular leukomalacia. Dev Neurosci. (2013) 35:182–96. 10.1159/000346682 23445614 PMC3764456

[15] Jantzie LL Corbett CJ Berglass J Firl DJ Flores J Mannix R. Complex pattern of interaction between in utero hypoxia-ischemia and intra-amniotic inflammation disrupts brain development and motor function. J Neuroinflammation (2014) 11:131. 10.1186/1742-2094-11-131 25082427 PMC4128546

[16] Skranes J Vangberg TR Kulseng S Indredavik MS Evensen KA Martinussen M. Clinical findings and white matter abnormalities seen on diffusion tensor imaging in adolescents with very low birth weight. Brain (2007) 130:654–66. 10.1093/brain/awm001 17347255

[17] Bora S Pritchard VE Chen Z Inder TE Woodward LJ. Neonatal cerebral morphometry and later risk of persistent inattention/hyperactivity in children born very preterm. J Child Psychol Psychiatry (2014) 55:828–38. 10.1111/jcpp.12200 24438003 PMC4065623

[18] van Tilborg E Heijnen CJ Benders MJ van Bel F Fleiss B Gressens P. Impaired oligodendrocyte maturation in preterm infants: potential therapeutic targets. Prog Neurobiol. (2016) 136:28–49. 10.1016/j.pneurobio.2015.11.002 26655283

[19] Baburamani AA Castillo-Melendez M Walker DW. VEGF expression and microvascular responses to severe transient hypoxia in the fetal sheep brain. Pediatr Res. (2013) 73:310–6. 10.1038/pr.2012.191 23222909

[20] Drury PP Davidson JO Bennet L Booth LC Tan S Fraser M. Partial neural protection with prophylactic low-dose melatonin after asphyxia in preterm fetal sheep. J Cereb Blood Flow Metab. (2014) 34:126–35. 10.1038/jcbfm.2013.174 24103904 PMC3887352

[21] Derrick M Luo NL Bregman JC Jilling T Ji X Fisher K. Preterm fetal hypoxia-ischemia causes hypertonia and motor deficits in the neonatal rabbit: a model for human cerebral palsy? J Neurosci. (2004) 24:24–34. 10.1523/JNEUROSCI.2816-03.2004 14715934 PMC6729589

[22] Derrick M Drobyshevsky A Ji X Tan S. A model of cerebral palsy from fetal hypoxia-ischemia. Stroke (2007) 38:731–5. 10.1161/01.STR.0000251445.94697.64 17261727

[23] Buser JR Segovia KN Dean JM Nelson K Beardsley D Gong X. Timing of appearance of late oligodendrocyte progenitors coincides with enhanced susceptibility of preterm rabbit cerebral white matter to hypoxia-ischemia. J Cereb Blood Flow Metab. (2010) 30:1053–65. 10.1038/jcbfm.2009.286 20068573 PMC2915781

[24] Greenwood K Cox P Mehmet H Penrice J Amess PN Cady EB Magnesium sulfate treatment after transient hypoxia-ischemia in the newborn piglet does not protect against cerebral damage. Pediatr Res. (2000) 48:346–50. 10.1203/00006450-200009000-00014 10960501

[25] Robertson NJ Faulkner S Fleiss B Bainbridge A Andorka C Price D. Melatonin augments hypothermic neuroprotection in a perinatal asphyxia model. Brain (2013) 136:90–105. 10.1093/brain/aws285 23183236

[26] Robertson NJ Kato T Bainbridge A Chandrasekaran M Iwata O Kapetanakis A. Methyl-isobutyl amiloride reduces brain Lac/NAA, cell death and microglial activation in a perinatal asphyxia model. J Neurochem. (2013) 124:645–57. 10.1111/jnc.12097 23171224

[27] Sizonenko SV Sirimanne E Mayall Y Gluckman PD Inder T Williams C. Selective cortical alteration after hypoxic-ischemic injury in the very immature rat brain. Pediatr Res. (2003) 54:263–9. 10.1203/01.PDR.0000072517.01207.87 12736386

[28] Fan LW Lin S Pang Y Lei M Zhang F Rhodes PG. Hypoxia-ischemia induced neurological dysfunction and brain injury in the neonatal rat. Behav Brain Res. (2005) 165:80–90. 10.1016/j.bbr.2005.06.033 16140403

[29] Robinson S Petelenz K Li Q Cohen ML Dechant A Tabrizi N. Developmental changes induced by graded prenatal systemic hypoxic-ischemic insults in rats. Neurobiol Dis. (2005) 18:568–81. 10.1016/j.nbd.2004.10.024 15755683

[30] Sizonenko SV Kiss JZ Inder T Gluckman PD Williams CE. Distinctive neuropathologic alterations in the deep layers of the parietal cortex after moderate ischemic-hypoxic injury in the P3 immature rat brain. Pediatr Res. (2005) 57:865–72. 10.1203/01.PDR.0000157673.36848.67 15774844

[31] Mazur M Miller RH Robinson S. Postnatal erythropoietin treatment mitigates neural cell loss after systemic prenatal hypoxic-ischemic injury. J Neurosurg Pediatr. (2010) 6:206–21. 10.3171/2010.5.PEDS1032 20809703 PMC3037962

[32] Tsuji M Ohshima M Taguchi A Kasahara Y Ikeda T Matsuyama T. A novel reproducible model of neonatal stroke in mice: comparison with a hypoxia-ischemia model. Exp Neurol. (2013) 247:218–25. 10.1016/j.expneurol.2013.04.015 23651512

[33] Rice JE III Vannucci RC Brierley JB. The influence of immaturity on hypoxic-ischemic brain damage in the rat. Ann Neurol. (1981) 9:131–41. 10.1002/ana.410090206 7235629

[34] Delcour M Russier M Xin DL Massicotte VS Barbe MF Coq JO. Mild musculoskeletal and locomotor alterations in adult rats with white matter injury following prenatal ischemia. Int J Dev Neurosci. (2011) 29:593–607. 10.1016/j.ijdevneu.2011.02.010 21382470

[35] Delcour M Olivier P Chambon C Pansiot J Russier M Liberge M. Neuroanatomical, sensorimotor and cognitive deficits in adult rats with white matter injury following prenatal ischemia. Brain Pathol. (2012) 22:1–16. 10.1111/j.1750-3639.2011.00504.x 21615591 PMC8028965

[36] Mizuno K Hida H Masuda T Nishino H Togari H. Pretreatment with low doses of erythropoietin ameliorates brain damage in periventricular leukomalacia by targeting late oligodendrocyte progenitors: a rat model. Neonatology (2008) 94:255–66. 10.1159/000151644 18784421

[37] Misumi S Ueda Y Nishigaki R Suzuki M Ishida A Jung CG. Dysfunction in motor coordination in neonatal white matter injury model without apparent neuron loss. Cell Transplant. (2016) 25:1381–93. 10.3727/096368915X689893 26564423

[38] Ueda Y Misumi S Suzuki M Ogawa S Nishigaki R Ishida A. Disorganization of oligodendrocyte development in the layer II/III of the sensorimotor cortex causes motor coordination dysfunction in a model of white matter injury in neonatal rats. Neurochem Res. (2017) 43:127–37. 10.1007/s11064-017-2352-3 28762105

[39] Ramanathan D Conner JM Tuszynski MH. A form of motor cortical plasticity that correlates with recovery of function after brain injury. Proc Natl Acad Sci USA. (2006) 103:11370–5. 10.1073/pnas.0601065103 16837575 PMC1544093

[40] Girgis J Merrett D Kirkland S Metz GA Verge V Fouad K. Reaching training in rats with spinal cord injury promotes plasticity and task specific recovery. Brain (2007) 130:2993–3003. 10.1093/brain/awm245 17928316

[41] Krajacic A Ghosh M Puentes R Pearse DD Fouad K. Advantages of delaying the onset of rehabilitative reaching training in rats with incomplete spinal cord injury. Eur J Neurosci. (2009) 29:641–51. 10.1111/j.1460-9568.2008.06600.x 19222562

[42] Ishida A Isa K Umeda T Kobayashi K Kobayashi K Hida H. Causal link between the cortico-rubral pathway and functional recovery through forced impaired limb use in rats with stroke. J Neurosci. (2016) 36:455–67. 10.1523/JNEUROSCI.2399-15.2016 26758837 PMC4710769

[43] Koyama Y Nishida T Tohyama M. Establishment of an optimised protocol for a Golgi-electron microscopy method based on a Golgi-Cox staining procedure with a commercial kit. J Neurosci Methods (2013) 218:103–9. 10.1016/j.jneumeth.2013.05.004 23721893

[44] Ishida A Misumi S Ueda Y Shimizu Y Cha-Gyun J Tamakoshi K Early constraint-induced movement therapy promotes functional recovery and neuronal plasticity in a subcortical hemorrhage model rat. Behav Brain Res. (2015) 2841:158–66. 10.1016/j.bbr.2015.02.022 25700666

[45] Paolicelli RC Bolasco G Pagani F Maggi L Scianni M Panzanelli P. Synaptic pruning by microglia is necessary for normal brain development. Science (2011) 333:1456–8. 10.1126/science.1202529 21778362

[46] Miyamoto A Wake H Moorhouse AJ Nabekura J. Microglia and synapse interactions: fine tuning neural circuits and candidate molecules. Front Cell Neurosci. (2013) 7:70. 10.3389/fncel.2013.00070 23720611 PMC3654203

[47] Kaur C Rathnasamy G Ling EA. Biology of microglia in the developing brain. J Neuropathol Exp Neurol. (2017) 76:736–53. 10.1093/jnen/nlx056 28859332

[48] Vilalta A Brown GC. Neurophagy, the phagocytosis of live neurons and synapses by glia, contributes to brain development and disease. FEBS J. (2017). 10.1111/febs.14323. [Epub ahead of print]. 29125686

[49] Young NA Vuong J Teskey GC. Development of motor maps in rats and their modulation by experience. J Neurophysiol. (2012) 108:1309–17. 10.1152/jn.01045.2011 22723681

[50] Jacobs KM Donoghue JP. Reshaping the cortical motor map by unmasking latent intracortical connections. Science (1991) 251:944–7. 2000496 10.1126/science.2000496

[51] Hu F Strittmatter SM. Regulating axon growth within the postnatal central nervous system. Semin Perinatol. (2004) 28:371–8. 10.1053/j.semperi.2004.10.001 15693393

[52] Wake H Moorhouse AJ Jinno S Kohsaka S Nabekura J. Resting microglia directly monitor the functional state of synapses *in vivo* and determine the fate of ischemic terminals. J Neurosci. (2009) 29:3974–80. 10.1523/JNEUROSCI.4363-08.2009 19339593 PMC6665392

[53] Kettenmann H Kirchhoff F Verkhratsky A. Microglia: new roles for the synaptic stripper. Neuron (2013) 77:10–18. 10.1016/j.neuron.2012.12.023 23312512

[54] Miyamoto A Wake H Ishikawa AW Eto K Shibata K Murakoshi H. Microglia contact induces synapse formation in developing somatosensory cortex. Nat Commun. (2016) 7:12540. 10.1038/ncomms12540 27558646 PMC5007295

[55] Johnston MV Hagberg H. Sex and the pathogenesis of cerebral palsy. Dev Med Child Neurol. (2007) 49:74–8. 10.1017/S0012162207000199.x 17209983

[56] Constable RT Ment LR Vohr BR Kesler SR Fulbright RK Lacadie C. Prematurely born children demonstrate white matter microstructural differences at 12 years of age, relative to term control subjects: an investigation of group and gender effects. Pediatrics (2008) 121:306–16. 10.1542/peds.2007-0414 18245422

[57] Sanches EF Arteni NS Nicola F Boisserand L Willborn S Netto CA. Early hypoxia-ischemia causes hemisphere and sex-dependent cognitive impairment and histological damage. Neuroscience (2013) 237:208–15. 10.1016/j.neuroscience.2013.01.066 23395861

[58] Sanches EF Arteni NS Scherer EB Kolling J Nicola F Willborn S. Are the consequences of neonatal hypoxia-ischemia dependent on animals' sex and brain lateralization? Brain Res. (2013) 1507:105–14. 10.1016/j.brainres.2013.02.040 23466455

[59] Ayoub AE Salm AK. Increased morphological diversity of microglia in the activated hypothalamic supraoptic nucleus. J Neurosci. (2003) 23:7759–66. 10.1523/JNEUROSCI.23-21-07759.2003 12944504 PMC6740605

[60] Perkins AE Piazza MK Deak T. Stereological analysis of microglia in aged male and female fischer 344 rats in socially relevant brain regions. Neuroscience (2018) 377:40–52. 10.1016/j.neuroscience.2018.02.028 29496632 PMC5882587

