# Supplementary figures and images for: Alterations of Both Dendrite Morphology and Weaker Electrical Responsiveness in the Cortex of Hip Area Occur Before Rearrangement of the Motor Map in Neonatal White Matter Injury Model
Source: Front Neurol. 2018 Jun 19;9:443. doi: 10.3389/fneur.2018.00443 (PMC6018077; doi:10.3389/fneur.2018.00443)

## Control (5 weeks of age)

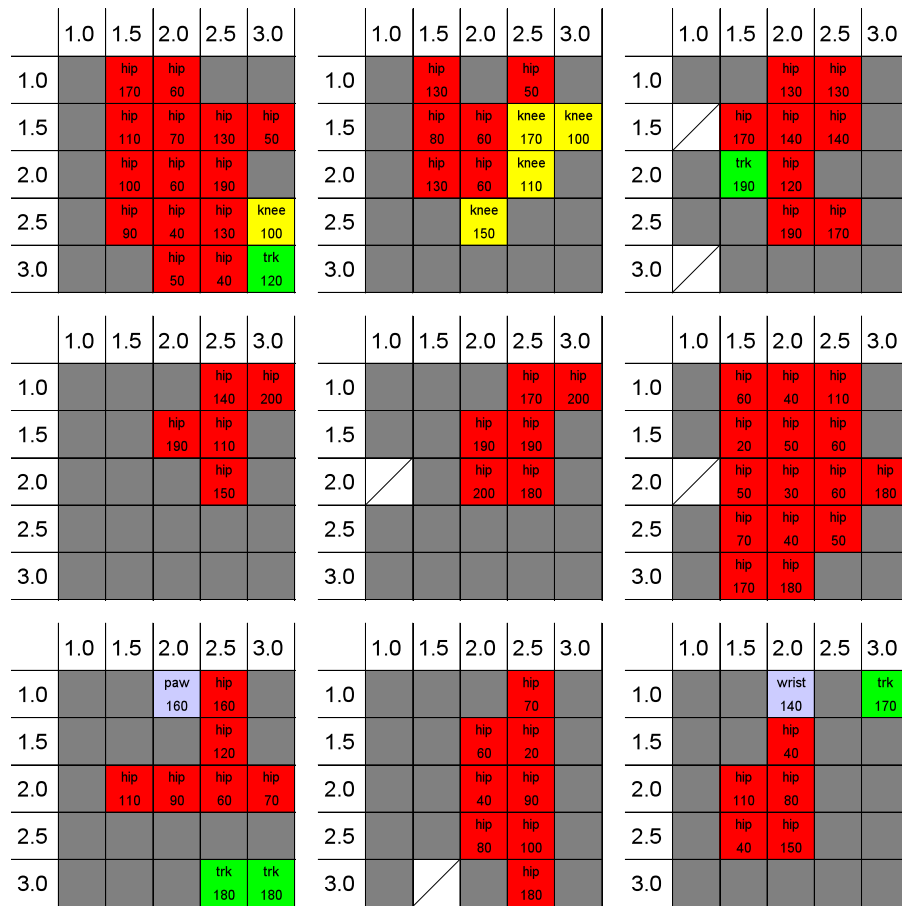

## WMI (5 weeks of age)

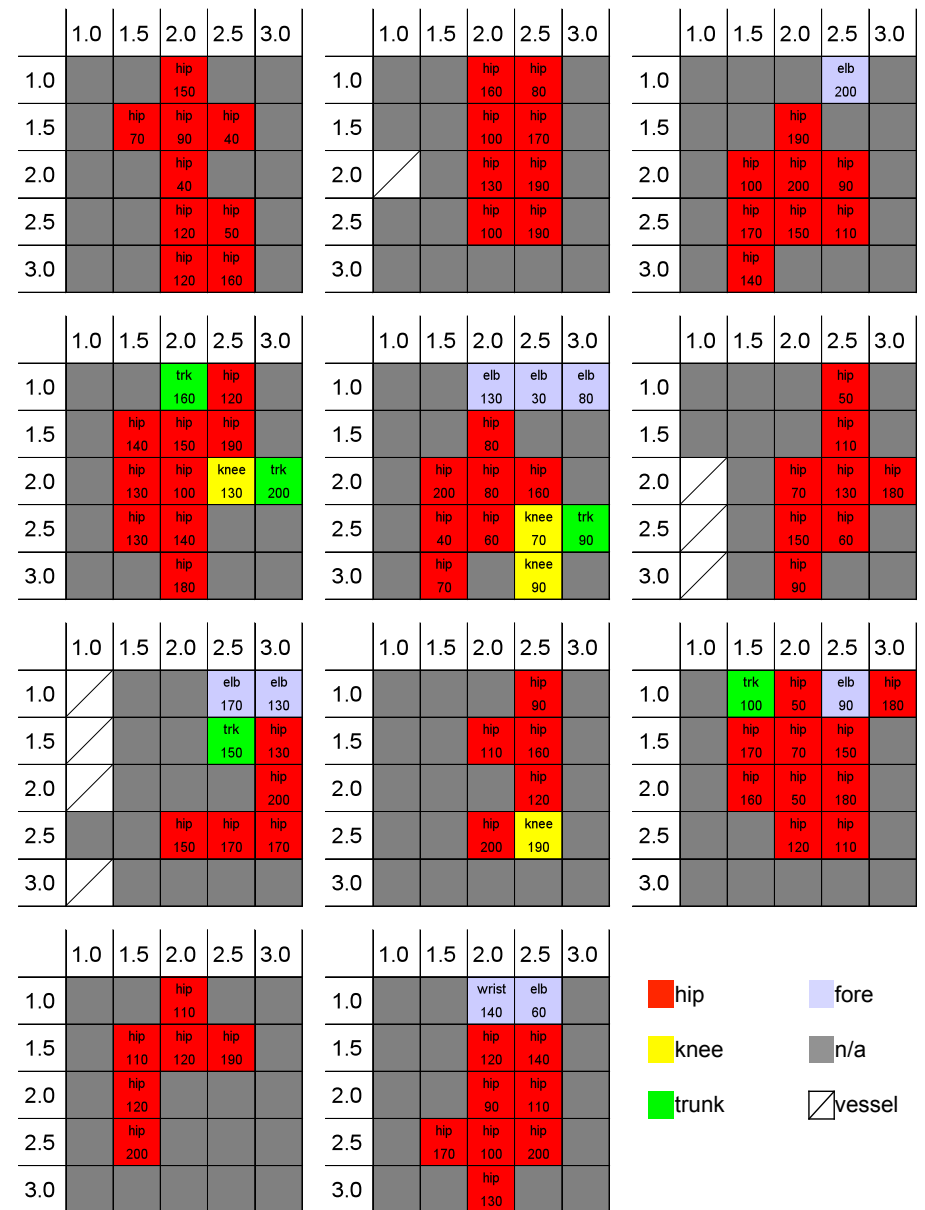

Supplement: Supplementary Figure 1 — Cortical motor map by ICMS at 5 weeks of age. Cortical maps at 5 weeks of age (n = 9 for control, n = 11 for neonatal WMI) are shown as a 5-colored code in the grid: twitch of the hip joint (red), knee joint (yellow), foot joint (light blue), and trunk (green), and non-responsive square (gray). The current threshold at each point is presented in each grid. [file Image_1.pdf]

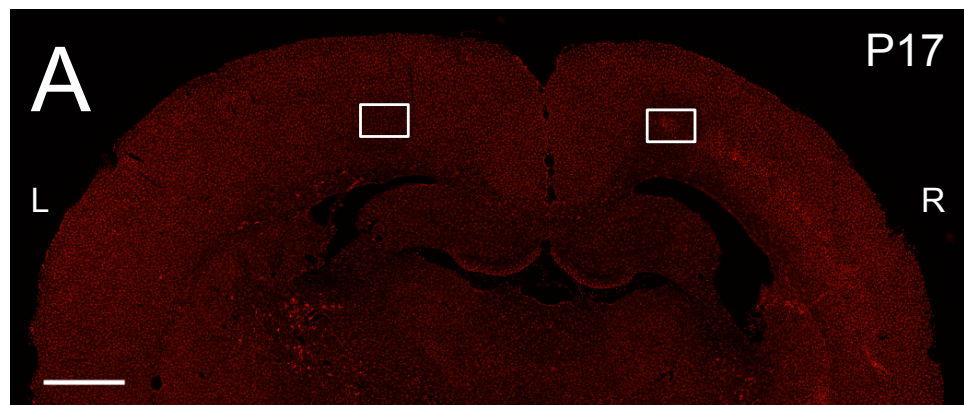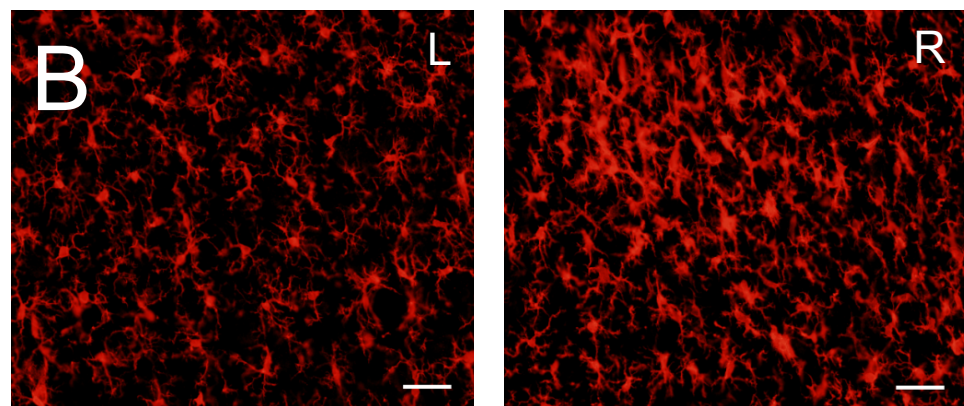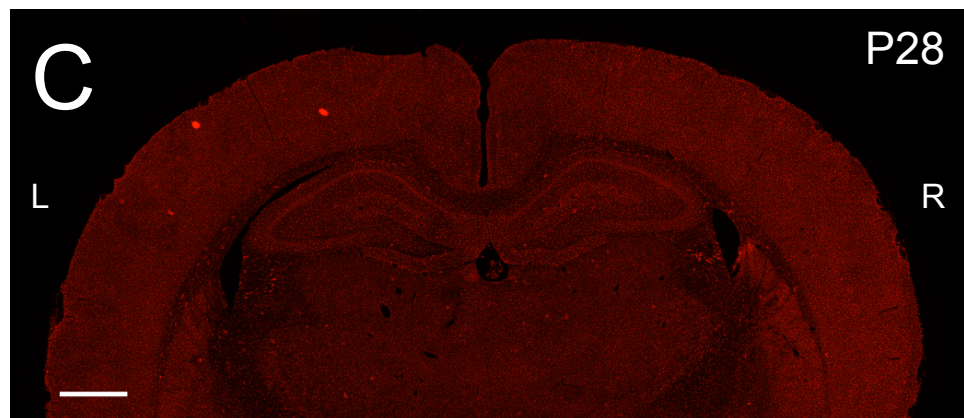

Supple Figure 2 Ueda et al

Supplement: Supplementary Figure 2 — Iba1 immunostaining. (A) Iba1 immunoreactivity was upregulated on the right H-I side of the cortex at P 17 (n = 4). (B) Appearance of Iba-1 positive microglia was increased in the right hemisphere (R) compared with the left side (L). (C) At P 28 (n = 4), the upregulation of immunoreactivity was not seen in either hemisphere. Error bars show 1 mm (A), and 40 μm (B). [file Image_2.pdf]
