# Supplementary material for: Alterations of Both Dendrite Morphology and Weaker Electrical Responsiveness in the Cortex of Hip Area Occur Before Rearrangement of the Motor Map in Neonatal White Matter Injury Model
Source: Front Neurol. 2018 Jun 19;9:443. doi: 10.3389/fneur.2018.00443 (PMC6018077; doi:10.3389/fneur.2018.00443)
Supplement: Supplementary Table 1 — Sholl analysis. [file Table_1.docx]

Supplemental Table 1 Sholl analysis

| Distance from cell body center (μm) | Number of cross sections | | p value |
| --- | --- | --- | --- |
|  | L | R |  |
| 20 | 6.7 ± 0.28 | 8.1 ± 0.28 | 0.00057 |
| 40 | 9.0 ± 0.32 | 12.3 ± 0.43 | 2.72E-07 |
| 60 | 8.9 ± 0.33 | 17.0 ± 0.59 | 6.94E-15 |
| 80 | 8.0 ± 0.35 | 19.2 ± 0.56 | 8.38E-21 |
| 100 | 7.7 ± 0.30 | 20.4 ± 0.52 | 6.76E-24 |
| 120 | 6.5 ± 0.25 | 19.6 ± 0.50 | 4.02E-24 |
| 140 | 6.2 ± 0.27 | 18.4 ± 0.32 | 4.19E-33 |
| 160 | 5.0 ± 0.18 | 17.3 ± 0.40 | 2.41E-26 |
| 180 | 4.7 ± 0.15 | 16.5 ± 0.36 | 2.65E-26 |

10 weeks of age

5 weeks of age

| Distance from cell body center (μm) | Number of cross sections | | p value |
| --- | --- | --- | --- |
|  | L | R |  |
| 20 | 7.0 ± 0.30 | 7.3 ± 0.21 | 0.42744 |
| 40 | 8.3 ± 0.31 | 10.4 ± 0.30 | 1.28E-05 |
| 60 | 8.2 ± 0.35 | 14.3 ± 0.35 | 5.82E-17 |
| 80 | 7.1 ± 0.37 | 17.7 ± 0.60 | 9.48E-19 |
| 100 | 6.8 ± 0.37 | 19.8 ± 0.77 | 1.25E-17 |
| 120 | 6.0 ± 0.26 | 18.6 ± 0.74 | 5.36E-17 |
| 140 | 5.4 ± 0.26 | 17.6 ± 0.64 | 7.19E-19 |
| 160 | 4.8 ± 0.25 | 16.1 ± 0.57 | 1.23E-19 |
| 180 | 4.6 ± 0.24 | 14.5 ± 0.54 | 9.64E-18 |
